# Ontological Organization and Bioinformatic Analysis of Adverse Drug Reactions From Package Inserts: Development and Usability Study

**DOI:** 10.2196/20443

**Published:** 2020-07-20

**Authors:** Xiaoying Li, Xin Lin, Huiling Ren, Jinjing Guo

**Affiliations:** 1 Institute of Medical Information Chinese Academy of Medical Sciences Beijing China

**Keywords:** ontology, adverse drug reactions, package inserts, information retrieval, natural language processing, bioinformatics, drug, adverse events, machine-understandable knowledge, clinical applications

## Abstract

**Background:**

Licensed drugs may cause unexpected adverse reactions in patients, resulting in morbidity, risk of mortality, therapy disruptions, and prolonged hospital stays. Officially approved drug package inserts list the adverse reactions identified from randomized controlled clinical trials with high evidence levels and worldwide postmarketing surveillance. Formal representation of the adverse drug reaction (ADR) enclosed in semistructured package inserts will enable deep recognition of side effects and rational drug use, substantially reduce morbidity, and decrease societal costs.

**Objective:**

This paper aims to present an ontological organization of traceable ADR information extracted from licensed package inserts. In addition, it will provide machine-understandable knowledge for bioinformatics analysis, semantic retrieval, and intelligent clinical applications.

**Methods:**

Based on the essential content of package inserts, a generic ADR ontology model is proposed from two dimensions (and nine subdimensions), covering the ADR information and medication instructions. This is followed by a customized natural language processing method programmed with Python to retrieve the relevant information enclosed in package inserts. After the biocuration and identification of retrieved data from the package insert, an ADR ontology is automatically built for further bioinformatic analysis.

**Results:**

We collected 165 package inserts of quinolone drugs from the National Medical Products Administration and other drug databases in China, and built a specialized ADR ontology containing 2879 classes and 15,711 semantic relations. For each quinolone drug, the reported ADR information and medication instructions have been logically represented and formally organized in an ADR ontology. To demonstrate its usage, the source data were further bioinformatically analyzed. For example, the number of drug-ADR triples and major ADRs associated with each active ingredient were recorded. The 10 ADRs most frequently observed among quinolones were identified and categorized based on the 18 categories defined in the proposal. The occurrence frequency, severity, and ADR mitigation method explicitly stated in package inserts were also analyzed, as well as the top 5 specific populations with contraindications for quinolone drugs.

**Conclusions:**

Ontological representation and organization using officially approved information from drug package inserts enables the identification and bioinformatic analysis of adverse reactions caused by a specific drug with regard to predefined ADR ontology classes and semantic relations. The resulting ontology-based ADR knowledge source classifies drug-specific adverse reactions, and supports a better understanding of ADRs and safer prescription of medications.

## Introduction

### Overview

Chemicals and drugs have made a great contribution to human health care. At the same time, they are rarely free from occasional adverse drug reactions (ADRs) [1], which are defined by the World Health Organization (WHO) as any noxious, unintended, and undesired effects of a drug that occur at doses used for the prevention, diagnosis, and treatment of a disorder. A significant number of ADRs occur each year. ADRs are the sixth leading cause of death worldwide, and the fourth primary cause of death in the United States and Canada, behind cardiovascular disease, malignant neoplasm, and stroke [2]. Although the actual incidence of ADRs is difficult to access precisely, it is known that ADRs have a considerable impact upon both health care and pharmaceutical manufacturers.

Package inserts (sometimes called patient information leaflets) are the primary official papers which accompany most prescribed drugs and over-the-counter medications. Although different countries have diverse requirements for the obligatory contents, the package inserts serve at least two main purposes. They contain informative details regarding the generic names of drugs, active ingredients, indication for use, instructions for use, special warnings, contraindications, and statistical values from clinical trials, including the percentage of people who had side effects, the types of side effects, and additional precautions. Furthermore, the package insert is an easy reference for physicians when prescribing medications, and can help them avoid prescribing drugs that may be contraindicated. The inserts also serve as an easy reference for patients. However, the informative package inserts are generally semistructured and cannot be understood easily by machines. As the number of newly licensed drugs increases, the demand for automatic technology for semantic integration and linkages, as well as bioinformatic analysis of the information (including ADRs) enclosed in package inserts, has become an urgent issue both in biomedical research and the pharmaceutical industry.

In information science, an ontology is the formal, explicit specification of a shared conceptualization of a domain [3]. Generally, biomedical ontologies not only represent the essential properties of biomedical entities and their correlations to other biomedical concepts, but also provide a standardized vocabulary and formalized knowledge source for the biomedical community. Hundreds of biomedical ontologies have been elaborately built to support scientific discovery and the analysis of biomedical data [4-8]. Moreover, systematically evaluated ontology is one of the two fundamental sources of background knowledge for artificial intelligence algorithms in biomedicine (the other is the knowledge graph).

**Figure 1:**
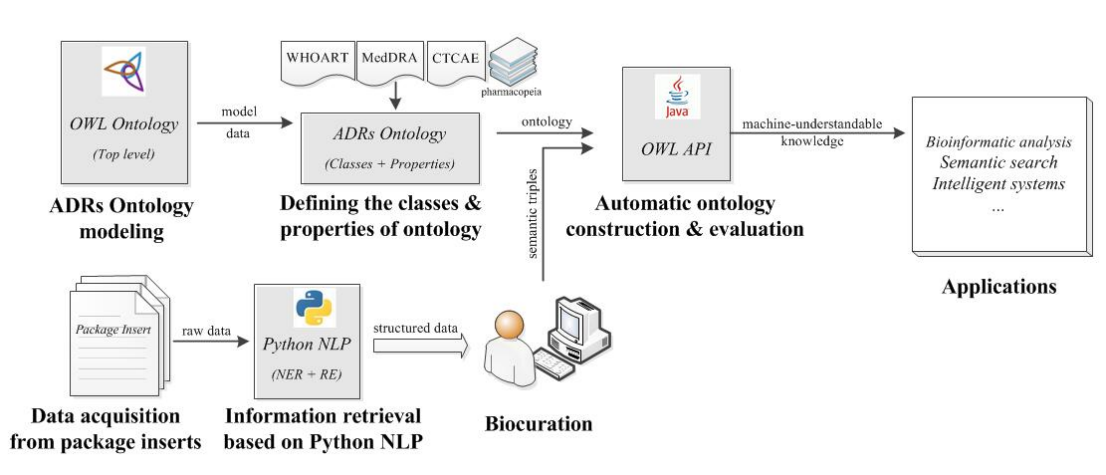
Study framework. ADRs: adverse drug reactions; API: application programming interface; CTCAE: Common Terminology Criteria for Adverse Events; MedDRA: Medical Dictionary for Regulatory Activities; NER: named entity recognition; NLP: natural language processing; OWL: Ontology Web Language; RE: relation extraction; WHOART: WHO Adverse Reactions Terminology.

In this paper, we propose an ontological organization of traceable ADR information extracted from licensed package inserts, which aims to provide machine-understandable knowledge for bioinformatics analysis, semantic retrieval, and intelligent clinical applications (Figure 1). This entails the following: (1) Present a generic ADR ontology model from two dimensions (and nine subdimensions) covering the essential ADR information and medication instructions. (2) Customize a Python natural language processing (NLP) method to automatically retrieve the identified information enclosed in package inserts. (3) Collect the approved package inserts of quinolone drugs and build a specialized ontology for algorithm verification and validation. (4) Bioinformatically analyze the adverse reactions caused by quinolones based on the obtained ADR ontology, and discuss potential applications including semantic retrieval and a clinical decision-making system.

### Related Works

#### Identification of ADRs

In recent decades, many studies have identified ADRs through diverse channels of information, including patient reports [9-14], electronic health records [15-18], PubMed literature [19,20], and social media [21-26]. Briefly, a patient report is a traditional source of information, where the users of a drug spontaneously report medication side effects to health authorities; electronic health records contain comprehensive medications and procedures as recorded by physicians; PubMed collects rich and up-to-date published clinical trials and other types of biomedical publications concerning drugs’ adverse reactions; and social media represents a new data source of patient experiences with drugs and could be characterized by its high volume and quick availability. Although patients, health professionals, research scientists, and even the public have increasingly contributed to ADR reporting, the role of pharmaceutical companies in reporting ADRs cannot be neglected; package inserts play a significant part in medication safety.

#### Ontologies of ADRs

Several studies about building ontologies for ADRs for different applications have already been carried out. For instance, the Adverse Drug Reaction Classification System (ADReCS) was developed as a comprehensive ADR ontology database, which enabled standardization and provided hierarchical classification of ADR terms for a molecular understanding of drug safety in the laboratory, and use in bioinformatics and systems biology for toxicological research [27]. Additionally, an ontology of ADRs (OADRs) was built to describe the semantics of ADR terms for automated signal generation in pharmacovigilance [28].

Adverse drug events (ADEs) refer to the injuries resulting from medical interventions related to drugs [1], which include medication errors, ADRs, allergic reactions, overdoses, and other events associated with the prescription, preparation, dispensation, or administration of medications. Therefore, research efforts concentrated on ontology-based representation and analysis of ADEs indirectly related to our work. Among them, the Ontology of Adverse Events (OAE) has recently garnered research attention; it represents numerous adverse events related to medical intervention, time at medical intervention, pathological bodily process, patient information (especially patient age), and other adverse event–related terms imported from existing ontologies, as well as clinical adverse event reports [29]. To better analyze adverse events related to vaccines and support safety studies of vaccines, the authors further expanded OAE and developed the Ontology of Vaccine Adverse Events (OVAE) by analyzing the adverse events recorded in the official packet inserts of licensed vaccines [30]. All the ontologies mentioned above provide a fundamental basis on which to conduct our study and will be compared with the proposed ADR ontology from several perspectives.

## Methods

### Ontology Modeling

**Figure 2:**
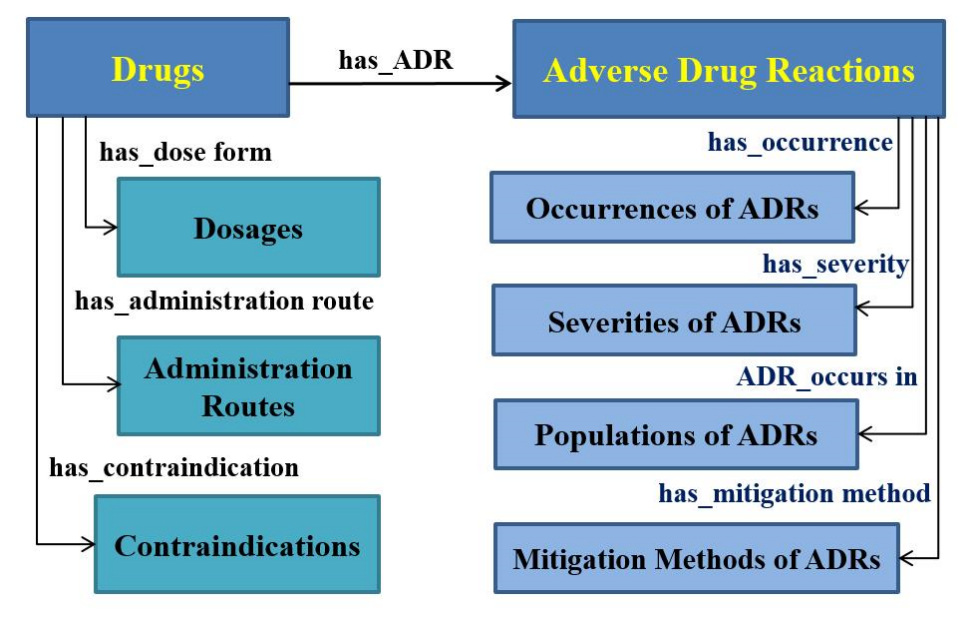
The generic model of ADRs ontology. ADRs: adverse drug reactions.

We built the ADR ontology using two dimensions: ADR information and drug-related medication instructions, which were further separated into nine subdimensions. The former covers the following: (1) adverse drug reactions, presenting diverse types of ADRs; (2) the occurrence of ADRs, describing the frequencies of ADRs after the administration of a drug in a population; (3) the severity of ADRs, describing a general measure of the subsequent risks of potential ADRs; (4) populations affected by specific ADRs, noting the individual human patients associated with an adverse reaction after the administration of a drug; and (5) the mitigation methods of ADRs, referring to any measure that shortens the duration of an adverse reaction or reduces its severity. The drug-related medication instructions contain generic information about rational drug use: (6) drug names, describing different pharmaceutical agents; (7) dosage forms of drugs, collecting the complete form of the pharmaceutical preparation used to administer the prescribed dose of medication; (8) administration routes of drugs, consisting of the various ways of administering a drug to a patient to allow the chemical to be absorbed into the blood and delivered to the target tissue; and (9) contraindications of drugs, predefining a condition or factor associated with a recipient that makes the use of a specific drug improper or inadvisable. These nine branches determine the fundamental concepts and classes of our ontology. Figure 2 demonstrates the nine classes of proposed ADR ontology as well as the eight semantic relations among them. The generic model will help to generate specialized ADR ontologies on request as illustrated in the next section.

### Classes of Ontology and Their Hierarchy and Properties

The biomedical terms denoting nine classes within the ADR ontology are much more comprehensive than shown in Figure 1. With reference to controlled biomedical vocabularies and well-developed pharmacopeia, we integrated many synonymous terms (both in English and Chinese) into various concepts and organized them in a hierarchical way. Generally, the WHO Adverse Reactions Terminology (WHOART) is reused to generate the ADR class and its hierarchy in four levels: system/organ classes (SOC), high level terms (HLT), preferred terms (PT), and included terms (IT). Among them, SOC and HLT consist of broad grouping terms, while PT represents more specific adverse reactions and IT are entry terms (synonyms) for PT. The large number of PT and IT as well as their synonyms from the Medical Dictionary for Regulatory Activities (MedDRA) will become the essential vocabulary to recognize the named entities of ADRs in package inserts. Furthermore, the Council for International Organization of Medical Science (CIMOS) has recommended five terms derived from different percentages to classify the occurrences of ADRs: Very Rare (<0.01%), Rare (≥0.01%, <0.1%), Uncommon (≥0.1%, <1%), Common (≥1%, <10%), and Very Common (≥10%). This forms the basis for the classification of ADR frequencies in our work. The Common Terminology Criteria for Adverse Events (CTCAE) is a standard classification and severity grading scale for adverse events in clinical trials and oncology settings. We adopted CTCAE for labeling the severities of ADRs with five levels: Grade 1 (mild), Grade 2 (moderate), Grade 3 (severe but not immediately life-threatening), Grade 4 (life-threatening) and Grade 5 (death caused by ADRs). The other classes that are closely related to the pharmaceutical agents and their medication instructions will be acquired and represented according to the classic pharmacopeia for ease of use.

In addition, two kinds of data properties will be incorporated into the ADR ontology, including a general description of the drugs (such as the active ingredient, injection excipients, drug specifications, antibacterial mechanism, interaction drugs); the key codes and definitions from referenced vocabularies containing ARecNO (the PT code) and the SOC code from WHOART, the MedDRA code, the NCIt code; and definitions from CTCAE. Furthermore, the origin of the package inserts will be recorded in the ontology’s annotation property for ontology data identification and traceability.

### Information Retrieval From Package Inserts

From the viewpoint of ontology construction, the ADR information and medication instructions extracted from package inserts will become the instances of object properties within the ADR ontology. Due to the large amount of data enclosed in package inserts, manual extraction would be a labor-intensive process. We developed a Python NLP-based algorithm to automatically retrieve ADRs and drug-related information, which consists of two steps: named entity recognition (NER) and relation extraction (RE).

Briefly, NER will recognize a string of text as an entity (eg, an adverse reaction) that is already defined in our ADR ontology. The Jieba word segmentation model implemented in Python is adopted to segment the words enclosed in package inserts, while the names of classes and their synonyms from the proposed ontology will function as a domain vocabulary to improve performance. RE is a process that determines whether two entities have a specific relationship (eg, the “has_ADR” causality between a particular drug and an adverse reaction). Since formal package inserts have already been separated into several titled sections, these titles will be used to implement the RE task. Specifically, the titles “ADVERSE REACTIONS” and “WARNINGS AND PRECAUTIONS” are converted into semantic relations about ADR information, while the medication instructions for a particular drug would be extracted from the sections titled “DOSAGE AND ADMINISTRATION,” “CONTRAINDICATIONS,” and “USE IN SPECIFIC POPULATIONS.”

The information automatically retrieved from package inserts will be passed to downstream biocuration for data identification. The major criteria of the manual review process emphasize the following two points: (1) whether the medication instructions and information about ADRs caused by a specific drug was accurate and complete, without missing data or mistakes; and (2) the frequency and severity of a drug-ADR triple must be explicit; vague descriptions were not recorded. Eventually, the human biocurated semantic information is used to build the ADR ontology.

### Automated Ontology Construction and Evaluation

The Ontology Web Language (OWL) is a widely used programing language for defining and instantiating web-based ontologies. It provides a machine-understandable schema to describe classes and their semantic relations in a specific domain. In this work, the OWL application programming interface, a Java interface and implementation for OWL, is used to build the ADR ontology automatically based on content data primarily obtained from package inserts and well-established vocabularies (eg, WHOART, MedDRA, CTCAE). Moreover, to ensure high-quality results, the ADR ontology will be evaluated and validated by checking the clarity, coherence, extendibility, minimal encoding bias, and minimal ontological commitment, which are the fundamental principles of building a domain ontology [31].

## Results

### Data Collection and Ontology Construction

Quinolone drugs have become commonly used antibacterial agents due to their strong and broad-spectrum antibacterial activity, as well as their rapid and complete absorption in humans. As quinolone usage increases, the risk of ADRs increases proportionally. According to the Annual Report of National ADR Monitoring in China, the number of adverse reactions and events from quinolones has continuously been the second-highest among antibacterial drugs. Therefore, the safe administration of quinolones is a serious matter that requires more attention.

After half a century of development, quinolones have evolved from the first generation to the fourth generation. We collected 165 specific drug names of quinolones from the China Pharmaceutical Reference and the National Essential Drugs List in China, and further organized them based on their generation. The electronic package inserts of these drugs were then downloaded from the National Medical Products Administration and Yaozhi drug database in China. Although these package inserts were written in Chinese, the Chinese and English synonyms of the class names defined in the proposed ADR ontology will enable information extraction based on a customized Python program. Finally, an ADR ontology for quinolones was automatically built and evaluated for further analysis.

### Ontology Statistics and Visualization

**Figure 3:**
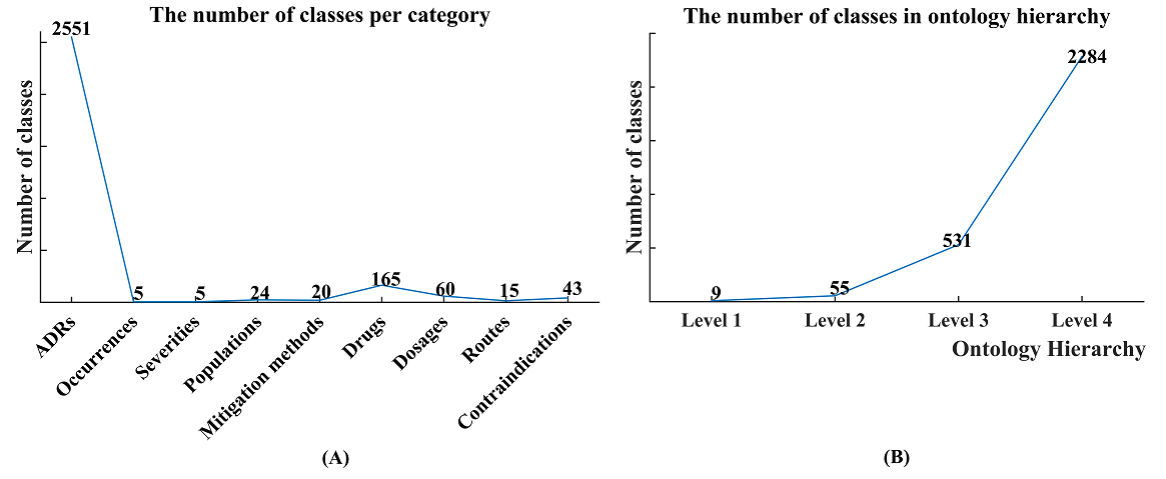
Statistics of the classes in ADRs ontology. ADRs: adverse drug reactions.

**Figure 4:**
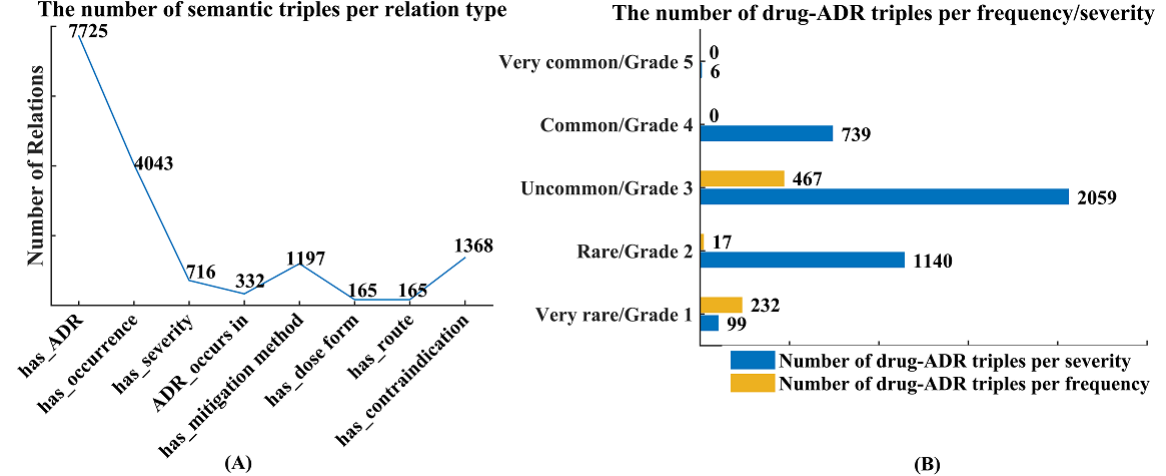
Statistics of the semantic relations in ADRs ontology. ADRs: adverse drug reactions.

Currently, the specialized ADR ontology covers a total of 2879 classes grouped into nine categories: ADRs and their occurrence, severity, population, and mitigation methods, as well as 165 drugs and their dosages, administration routes, and contraindications (Figure 3A). These classes were deeply divided into subclasses on the basis of biomedical concepts and arrayed hierarchically from most general to most specific in up to 4 levels, with the abovementioned nine categories at the top (Level 1) of the ontology hierarchy (Figure 3B). Furthermore, the obtained ADR ontology also includes eight types and 15,711 nonredundant semantic triples (Figure 4A) extracted from package inserts, where the causalities between ADRs and quinolones (with the type label “has_ADR”) account for a large proportion (n=7725, 49.17%). Among the 7725 identified drug-ADR triples, 4043 (52.34%) and 716 (9.27%) explicitly stated the frequency and severity, respectively. The statistics of drug-ADR triples by frequency and severity suggest that most ADRs caused by quinolones (n=4037, 52.26%) occur at a low frequency (<10%), and none of them are life-threatening (Figure 4B).

**Figure 5:**
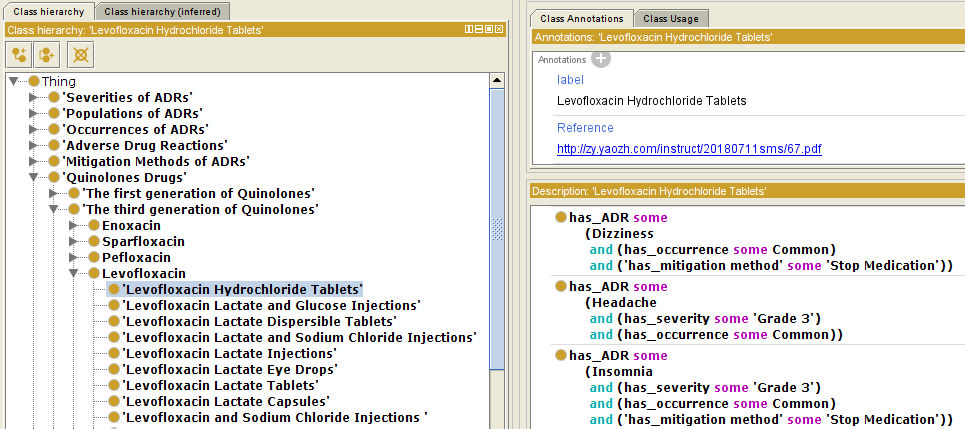
Ontology visualization using levofloxacin hydrochloride tablets as an example. ADRs: adverse drug reactions.

Figure 5 demonstrates how the proposed ontology organized the ADR information enclosed in the package insert, using the Protégé OWL editor for ontology visualization. Briefly, levofloxacin hydrochloride tablets have been reported to account for different types of ADRs (including dizziness, headache, and insomnia) that have already been defined in our ontology. For each adverse reaction, it is likely that the frequency, severity, and mitigation method are informed by the patient population. Therefore, we used four semantic relation types (“has_occurrence,” “has_severity,” “has_mitigation method,” and “has_population”) to link information associated with a particular adverse reaction caused by a specific drug (the lower right section of Figure 5). The “AND” clause is adopted to combine multiple semantic triples into a composite knowledge unit. It is worth mentioning that we extracted ADR information from the explicitly stated content in package inserts; thus, some of the four types linked to a given drug-ADR triple may not be obtained due to a missed or implicit description. To enable knowledge tracing, the package insert citation was recorded as a referenced annotation property (the upper right section of Figure 5). In addition, the Internationalized Resource Identifier (IRI) [32], which is the unique identifier for ontology sharing and reuse around the world, was customized as “ADR+six numbers” within the proposed ADR ontology. For instance, the IRI for the levofloxacin hydrochloride tablets used in the example is ADR000572.

### Comparison With Existing Ontologies

We compared our work with four other ontologies (described in the Introduction) in terms of the semantic relations defined in the proposed ADR ontology (Table 1), since they are the essential knowledge unit for the wide utility of an ontology. The proposed ontology represents a complete set of information pertaining to ADRs containing drug-ADR triples and the associated frequency, severity, and mitigation methods. Moreover, the explicit medication instructions (eg, the dosage form, route, and contraindication) extracted from the package insert of a particular drug support physicians in guiding patients in the safe, effective, and rational use of drugs, as an adverse reaction can occur when using the prescribed dosage.

**Table 1:** Comparison with existing ontologies.

Semantic relations of the proposed ADR^a^ ontology	Definition	Adverse Drug Reaction Classification System	Ontology of ADRs	Ontology of Adverse Events	Ontology of Vaccine Adverse Events
has_ADR	The adverse reaction caused by a specific drug.	Yes	Yes	Yes	Yes
has_occurrence	The occurrence frequency of an adverse reaction caused by a specific drug.	Yes	No	Yes	Yes
has_severity	The severity of an adverse reaction caused by a specific drug.	Yes	No	Yes	Yes
ADR_occurs in	The population in which an adverse reaction occurs.	No	No	Yes	Yes
has_mitigation method	Any measure that shortens the duration of an adverse reaction or reduces its severity.	No	No	No	No
has_dose form	The form of a dosage of a specific drug.	No	No	No	No
has_administration route	The prescribed way of administering a drug to a patient.	No	No	No	Yes
has_contraindication	A predefined condition or factor associated with a recipient that makes the use of a specific drug improper or inadvisable.	No	No	No	No

^a^ADR: adverse drug reaction.

### Bioinformatic Analysis of the Identified ADRs Caused by Quinolones

The ontology of ADRs caused by quinolones consists of 7725 drug-ADR triples retrieved from 165 package inserts. After duplicate removal, 331 ADRs were identified to be caused by quinolones. Table 2 lists the major ADRs from two dimensions: the quinolone generation and active ingredient. Since topical drugs have fewer ADRs than oral and intravenous ones due to their administration route, we concentrated on the comparison of quinolone drugs that are only administered by the oral and intravenous routes. After bioinformatic analysis, there were three important points that could be summarized as follows: (1) Levofloxacin (third generation) and gatifloxacin (fourth generation) induce a significant number of ADRs (n=139, 43.57% and n=122, 38.24%, respectively), and many drugs are made from these ingredients (n=21, 17.21% and n=20, 16.39%, respectively). (2) The highest numbers of ADRs are caused by ciprofloxacin (n=221, 69.28%) and enoxacin (n=156, 48.90%), respectively. (3) There are relatively few ADRs associated with nalidixic acid (n=21, 6.56%) and pipemidic acid (n=11, 3.45%), which implies that they are comparatively safe in terms of known adverse effects. In future work, we will investigate the ADR differences related to drug dosages.

**Table 2:** Identified adverse drug reactions of quinolones, excluding topical drugs (N=122).

Quinolone generation and ingredient		Drugs, n (%)		Drug-ADRs^a^ (n=7563), n (%)		ADRs (n=319), n (%)		Major ADRs		Typical dosage form(s)		Major routes	
**First generation**													
	Nalidixic acid		1 (0.82)		21 (0.28)		21 (6.56)		Nausea, vomiting, diarrhea, abdominal pain		Tablet		Oral
**Second generation**													
	Pipemidic acid		3 (2.46)		33 (0.44)		11 (3.45)		Nausea, eructation, abdominal pain		Granules, capsule, tablet		Oral
**Third generation**													
	Enoxacin		6 (4.92)		335 (4.43)		156 (48.90)		Skin rash, dizziness, abdominal pain		Tablet, capsule, injectable		Oral, intravenous
	Sparfloxacin		5 (4.10)		235 (3.11)		47 (14.73)		Headache, dizziness, insomnia, anemia		Tablet, capsule, granules		Oral
	Pefloxacin		5 (4.10)		150 (1.98)		35 (10.97)		Anaphylaxis, convulsion, tremor		Tablet, capsule, injectable		Oral, intravenous
	Levofloxacin		21 (17.21)		1592 (21.05)		139 (43.57)		Anaphylaxis, insomnia, dizziness		Tablet, capsule, injectable		Oral, intravenous
	Tolfloxacin		1 (0.82)		26 (0.34)		26 (8.15)		Fatigue, anorexia, erythematous rash		Capsule		Oral
	Fleroxacin		8 (6.56)		210 (2.78)		47 (14.73)		Nausea, vomiting, headache, dizziness		Tablet, capsule, injectable		Oral, intravenous
	Ofloxacin		9 (7.38)		303 (4.01)		87 (27.27)		Anaphylaxis, itching, skin rash		Tablet, capsule, injectable		Oral, intravenous
	Lomefloxacin		12 (9.84)		474 (6.27)		85 (26.65)		Itching, skin rash, headache, nausea		Tablet, capsule, injectable		Oral, intravenous
	Ciprofloxacin		8 (6.56)		684 (9.04)		221(69.28)		Skin rash, itching, diarrhea, hematuria		Tablet, capsule, injectable		Oral, intravenous
	Rufloxacin		2 (1.64)		68 (0.90)		34 (10.66)		Skin rash, insomnia, lethargy, convulsion		Tablet, capsule		Oral
	Norfloxacin		9 (7.38)		199 (2.63)		35 (10.97)		Itching, skin rash, abdominal pain		Tablet, capsule, injectable		Oral, intravenous
**Fourth generation**													
	Gatifloxacin		20 (16.39)		2270 (30.01)		122 (38.24)		Headache, vision disorder, dysgeusia		Tablet, capsule, injectable		Oral, intravenous
	Gemifloxacin		1 (0.82)		106 (1.40)		106 (33.22)		Skin rash, nausea, urticaria, diarrhea		Tablet		Oral
	Balofloxacin		2 (1.64)		66 (0.87)		33 (10.34)		Itching, thirst, hypesthesia, headache		Tablet, capsule		Oral
	Pazufloxacin		3 (2.46)		180 (2.38)		60 (18.81)		Skin rash, jaundice, myalgia, diarrhea		Injectable		Intravenous
	Prulifloxacin		3 (2.46)		189 (2.50)		63 (19.75)		Eructation, dyspnea, hypotension		Tablet, capsule		Oral
	Moxifloxacin		3 (2.46)		420 (5.55)		141 (44.20)		Fatigue, constipation, rupture of tendon		Tablet, injectable		Oral, intravenous

^a^ADR: adverse drug reaction.

The 331 ADRs could be further classified into 18 categories that we defined in the ontology (Table 3). Most organs are involved in ADRs. Skin reactions (eg, itching, skin rash) are the most common reactions and are linked to 141 drugs (85.45%), followed by nervous system reactions (eg, headache, hypertonia), which are associated with 127 agents (76.97%). Moreover, the least frequently reported adverse reactions are those of genital organs and the application site. This may be due to the difficulty in detecting genital organ diseases and the minor impact of injection site reactions.

**Table 3:** Categorized adverse drug reactions of quinolones (N=165).

Category of adverse drug reaction	Associated drugs, n (%)	Possible adverse drug reactions
Skin reactions	141 (85.5)	Itching, skin rash, photosensitive reaction, erythema multiforme, increased sweating
Nervous system reactions	127 (77.0)	Headache, hypertonia, convulsion, coma, paresthesia, vertigo, tremor
Immune reactions and infections	123 (74.6)	Candidiasis, anaphylaxis, angioneurotic edemas, anaphylactic shock, facial edema
Gastrointestinal reactions	122 (73.9)	Nausea, vomiting, abdominal pain, diarrhea, stomatitis, constipation, xerostomia
Generalized reactions	120 (72.7)	Fever, fatigue, syncope, chest pain, shivering, edema, oral edema, discomfort
Mental disorders	119 (72.1)	Sleeplessness, personality disorders, hallucinations, depression, agitation, anxiety
Liver and gallbladder diseases	117 (70.9)	Alanine transaminase (ALT) elevation, jaundice, alkaline phosphatase increased, liver failure, bilirubinemia
Urinary diseases	111 (67.3)	Hematuria, urinary incontinence, dysuria, crystalluria, interstitial nephritis
Musculoskeletal diseases	105 (63.6)	Arthritis, arthralgia, muscle weakness, myalgia, rupture of tendon, bone pain
Hematological diseases	103 (62.4)	Eosinophilia, leukopenia, granulocytopenia, pancytopenia, lymphadenopathy
Vascular, hemorrhagic, and coagulation diseases	102 (61.8)	Elevated international normalized ratio (INR) value, purpura, phlebitis, vasculitis, vasodilatation, flushing, thrombocytosis
Cardiovascular diseases	80 (48.5)	Prolonged QT interval, hypotension, ventricular tachycardia, palpitation, bradycardia
Respiratory system diseases	71 (43.0)	Pulmonary infiltration, bronchial spasm, asthma, laryngeal edema, dyspnea
Metabolic and nutrition diseases	63 (38.2)	Hypoglycemia, electrolytes abnormality, diabetes mellitus, hyperglycemia
Vision diseases	62 (37.6)	Xerophthalmia, eye pain, conjunctivitis, diplopia, photophobia, abnormal vision
Auditory, vestibular, and sensory diseases	62 (37.6)	Tinnitus, hypoacusis, deafness, taste disorders, parosmia, earache, ageusia
Genital organ diseases	48 (29.1)	Vaginitis, epididymitis, orchitis, dysmenorrhea, uterine hemorrhage
Application site reactions	19 (11.5)	Injection site reaction, injection site itching, infusion site reaction, injection site pain

Table 4 lists the top 10 ADRs caused by quinolones and shows that itching and skin rash are listed in nearly 80% of currently licensed quinolone package inserts in China. Although alanine transaminase (ALT) elevation and phlebitis frequently occur after the administration of 3 drugs made of pazufloxacin mesylate, other adverse reactions caused by quinolones are usually infrequent (<10%). All of the reported ADRs have a severity of Grade 1 (mid) to Grade 3 (severe but not immediately life-threatening), and stopping medication is generally recommended as the mitigation method after the detection of an adverse reaction. Additionally, quinolone drugs are administered to patients at various dosages and through different routes (Table 2). Finally, analysis of contraindication data found that the top 5 specific populations are those who are allergic to quinolones, pregnant women, teenagers, infants, and patients with central nervous system diseases.

**Table 4:** The 10 most commonly reported adverse drug reactions of quinolones (N=165).

Adverse drug reaction	Related drugs, n (%)	Category of adverse drug reaction
Itching	132 (80.0)	Skin diseases
Skin rash	131 (79.4)	Skin diseases
Headache	126 (76.4)	Nervous system diseases
Nausea	119 (72.1)	Gastrointestinal diseases
Abdominal pain	117 (70.9)	Gastrointestinal diseases
Vomiting	116 (70.3)	Gastrointestinal diseases
Diarrhea	114 (69.1)	Gastrointestinal diseases
Insomnia	110 (66.7)	Mental disorders
Fever	104 (63.0)	Generalized diseases
Photosensitive reaction	100 (60.6)	Skin diseases

## Discussion

### Major Applications of ADR Ontology

The ADR ontology proposed in this study has two major applications. The first is the semantic retrieval system that can use the ADR knowledge to integrate various external sources of information. Since the primary ADR terms and their key codes were reused from WHOART and MedDRA, it should be straightforward to integrate our ontology with these controlled vocabularies, as well as other medical terminologies (eg, Logical Observation Identifiers Names and Codes [LOINC], Systematized Nomenclature of Medicine-Clinical Terms [SNOMED CT]), for semantic knowledge retrieval by providing formally represented ADR information. Another major application is intelligent clinical decision-making support. The proposed ADR ontology provides machine-understandable knowledge, which could be used by artificial intelligence algorithms in biomedicine. To pursue clinical and therapeutic approaches to optimal disease management and rational drug use, it is useful for a physician treating a specific disorder to know all the identified adverse reactions induced by the drugs prescribed for patients with that condition. Manually reading the package inserts to find the ADR information and medication instructions is laborious and time-consuming, as the number of newly approved drugs and reported ADRs increases every year. Conversely, our ontology will aid in the development of an intelligent clinical decision-making system, which would positively affect the drug prescribing patterns of physicians and potentially have a significant socioeconomic impact.

### Conclusions

We have shown that the ADR ontology can be used to formally represent and organize the ADR information and medication instructions enclosed in official drug packages. In addition, it can provide machine-understandable knowledge for bioinformatic analysis. By collecting 165 package inserts of quinolone drugs, a specialized ADR ontology was built to classify various identified ADRs caused by quinolones. Systematic analysis of the obtained ontology data improves the deep recognition of drug-specific ADRs, making it possible to intelligently guide safe drug use and benefit human health.

The proposed ADR ontology can be generalized to organize the ADR information from other channels, not just package inserts. Electronic health records, in which the ADR data are frequently reported, will be acquired for the enrichment of the ADR ontology in the near future.

## Abbreviations

ADE: adverse drug event
ADR: adverse drug reaction
CIMOS: Council for International Organization of Medical Science
CTCAE: Common Terminology Criteria for Adverse Events
HLT: high level terms
IRI: Internationalized Resource Identifier
IT: included terms
LOINC: Logical Observation Identifiers Names and Codes
MedDRA: Medical Dictionary for Regulatory Activities
NER: named entity recognition
NLP: natural language processing
OWL: Ontology Web Language
PT: preferred terms
RE: relation extraction
SNOMED CT: Systematized Nomenclature of Medicine-Clinical Terms
SOC: system/organ classes
WHO: World Health Organization
WHOART: WHO Adverse Reactions Terminology
